# Psychometric validation and meaningful change thresholds of the Skindex-10 questionnaire and 5-D Itch scale for assessing itch in patients with chronic kidney disease-associated pruritus

**DOI:** 10.1186/s41687-025-00973-3

**Published:** 2025-11-27

**Authors:** Margaret K. Vernon, Catherine Munera, Robert H. Spencer, Warren Wen, Frédérique Menzaghi

**Affiliations:** 1 Evidera, Bethesda, MD USA; 2 Cara Therapeutics, Inc, 400 Atlantic Street, Suite 500, Stamford, CT 06901 USA

**Keywords:** Chronic kidney disease, Numerical rating scale, Patient global impression of worst itch severity, Patient-reported outcome measures, Pruritus, Psychometrics, Skindex-10, 5-D Itch

## Abstract

**Background:**

The Skindex-10 questionnaire and 5-D Itch scale are patient-reported outcome measures used to evaluate the itch intensity and impact of chronic pruritus on patients’ quality of life (QoL). These measures may be appropriate for evaluating the efficacy of anti-pruritic treatment in patients with chronic kidney disease-associated pruritus (CKD-aP). This study evaluated the psychometric validity and meaningful within-patient change (MWPC) thresholds of these two measures in patients with moderate-to-severe CKD-aP undergoing hemodialysis.

**Methodology:**

Content validity interviews for the Skindex-10 were conducted in 23 patients. Psychometric properties of the Skindex-10 questionnaire and 5-D Itch scale were assessed using data collected from a phase 2 randomized controlled trial of an anti-pruritic treatment in patients with moderate-to-severe CKD-aP. As part of the trial, the patients (*N* = 174) had completed the Skindex-10 and 5-D Itch measures at baseline (Day 1, pre-treatment); Weeks 2, 4, 6; and end of treatment (Week 8). Anchor-based methods were used to determine MWPC score thresholds, which were verified in two very similar, larger phase 3 cohorts (*N* = 378 and *N* = 471).

**Results:**

Content validity interview participants considered the Skindex-10 questionnaire to be straightforward, relevant, and comprehensive. Scores from both measures showed moderate-to-strong internal consistency (Cronbach’s α ≥ 0.74), and moderate test-retest reliability (intraclass correlation coefficients ≥ 0.67, Week 2 vs. Week 4) for patients stable on the similar Patient Global Impression of Worst Itch Severity measure. Construct validity analyses indicated that total scores and most domains of the Skindex-10 and 5-D Itch significantly correlated with other measures of itch. Skindex-10 and 5-D Itch total scores were also significantly different (*P* ≤ 0.0015) between distinct groups created based on conceptually related pruritus measures (known-groups validity). Anchor-based analyses indicated that a reduction from baseline of ≥ 15 points on the 0–60 scale for Skindex-10 total score, and of ≥ 5 points on the 5–25 scale for 5-D Itch total score, represented appropriate clinically meaningful within-patient changes.

**Conclusions:**

The Skindex-10 and 5-D Itch showed good psychometric properties for measuring itch intensity and health-related quality of life in patients with moderate-to-severe CKD-aP. These results support their use in evaluating treatment efficacy in this patient population.

**Supplementary Information:**

The online version contains supplementary material available at 10.1186/s41687-025-00973-3.

## Abstract (unstructured)

Patients with advanced chronic kidney disease often suffer from intense and bothersome itching, which can greatly lower their quality of life. To determine whether new treatments for itching are effective, clinicians need to assess if the itching and its impact on patients’ lives improve over time. Only the patients themselves can accurately report how severe the itching is and how it affects their daily lives. Patients can fill out questionnaires and scales to provide this information, but these tools need to be tested to ensure they work well for this specific group of patients. This study tested two tools for measuring how itching affects the quality of life in patients with advanced chronic kidney disease. These tools are called the “Skindex-10 questionnaire” and the “5-D Itch scale”. Using data from previous clinical trials of an anti-itch treatment in these patients, we found that both tools gave reliable results in test-retest experiments, and the results matched well with other established methods for measuring itch impact. For each measure, we also calculated how much change represents a meaningful improvement for patients with chronic kidney disease who had moderate-to-severe itching at the start of treatment. These findings support the use of these two tools to assess whether treatments for itching truly help patients with chronic kidney disease.

**Supplementary Information:**

The online version contains supplementary material available at 10.1186/s41687-025-00973-3.

## Background

Up to 40% of patients with chronic kidney disease (CKD) experience moderate-to-severe pruritus, characterized by generalized and persistent itching that often leads to considerable skin damage, chronic lesions, and cutaneous infections due to uncontrollable scratching [1–4]. Moderate-to-severe chronic kidney disease-associated pruritus (CKD-aP) is associated with reduced quality of life, poor sleep quality, depression, social anxiety, and increased risk of death [1–3, 5, 6]. Until recently, therapy for CKD-aP was limited to off-label treatments in most countries.

Because itch is a subjective experience, clinical trials must use patient-reported outcome (PRO) measures to evaluate how antipruritic treatments modify the intensity of itch and its impact on patients’ quality of life. The psychometric properties of PRO measures of itch should be validated in the population under study to ensure their capability and accuracy in assessing treatment effects. This validation is also required by best practices and US Food and Drug Administration evidentiary standards to support claims in approved medical product labeling [7]. Furthermore, to interpret clinical study results, it is important to know the magnitude of change on the PRO measure that represents a meaningful benefit to patients (i.e., a ‘responder definition’) since the start of anti-pruritic treatment.

We previously determined the psychometric properties and threshold of meaningful within-person change (MWPC) for the Worst Itching Intensity Numerical Rating Scale (WI-NRS) in patients with CKD-aP [8, 9]. The WI-NRS was one of the measures used to evaluate the efficacy of difelikefalin, an anti-pruritic treatment, in the drug’s phase 2 and pivotal phase 3 trials conducted in patients with moderate-to-severe CKD-aP [10–13]. The Skindex-10 questionnaire and 5-D Itch scale were additional PRO measures used to evaluate efficacy and change in quality of life in these clinical trials [10–13]; however, the psychometric properties of these measures have not been confirmed in patients with CKD-aP. Moreover, the threshold score change that indicates a within-patient meaningful improvement has not been determined in this population for either instrument.

The Skindex-10 questionnaire, a modification of the widely used Skindex-16 instrument, was developed specifically for CKD-aP as a disease-specific measure of health-related quality of life (HRQoL) [3]. Patients are asked to answer 10 questions on how bothered they have been by certain items related to their itch over the past week. Within each question, they fill in one of seven circles numbered from 0 (labeled with the anchor phrase “never bothered”) to 6 (labeled as “always bothered”). The total score is the sum of the numeric value of each answered question and ranges from 0 to 60. The Skindex-10 is negatively scored, meaning that higher scores reflect worse HRQoL. The total score is subdivided into three domain scores: disease domain (questions 1 to 3 with score range of 0 to 18); mood/emotional distress domain (questions 4 to 6 with score range of 0 to 18); and social functioning domain (questions 7 to 10 with score range of 0 to 24). To date, only the instrument’s test-retest reliability and correlations with other PRO measures of itch intensity and HRQoL have been assessed within a CKD-aP population [3].

The 5-D Itch scale was developed as a brief but multidimensional questionnaire designed to evaluate pruritus of various origins in clinical trials [14]. Patients are asked to assess their itch symptoms according to five dimensions (degree, duration, direction, disability, and distribution) based on their experiences over the past 2 weeks. The first three domains – degree, duration, and direction – are measured by a five-point Likert scale with higher scores reflecting worse symptoms of itch. The fourth domain, disability, includes four items that assess the impact of itching on daily activities – sleep, leisure/social activities, housework/errands, and work/school – with each item scored on a five-point Likert scale with 5 representing the most severe impact. The disability domain score is determined as the highest score of any of the four items. For the fifth domain, distribution, the number of affected body parts is tallied (potential sum 0–16) and the sum is sorted into five scoring bins: sum of 0–2 = score of 1, sum of 3–5 = score of 2, sum of 6–10 = score of 3, sum of 11–13 = score of 4, and sum of 14–16 = score of 5. The scores of each of the five domains are calculated separately and then summed together to obtain a total 5-D score. The total score can range from 5 (no pruritus) to 25 (most severe pruritus). The scale has undergone some quantitative validation [14], mostly in populations with chronic pruritus due to other causes (e.g., chronic liver disease, skin diseases, and burns), and has been shown to be sensitive to changes in pruritus over time [14–16]. Content validity of the 5-D Itch scale was initially established in patients participating in a trial for a treatment of cholestatic pruritus, although patients with CKD were subsequently included in the original development of the instrument with respect to its validity, reliability, and response to change [14].

The aim of this study was to evaluate the psychometric properties of the Skindex-10 questionnaire and 5-D Itch scale in hemodialysis patients with moderate-to-severe CKD-aP and to identify thresholds of MWPC for these PRO measures.

## Methods

### Content validity methods

Content validity of the Skindex-10 questionnaire was evaluated through one-on-one qualitative interviews with 23 hemodialysis patients with CKD-aP of any severity. The recruitment, eligibility criteria, conduct, and analysis of these qualitative interviews has been previously described [17]. The interviews were conducted in two parts – a concept elicitation (reported previously [17]) and a cognitive debriefing on the Skindex-10. The concept elicitation verified that the concepts evaluated by the Skindex-10 are relevant for the patient population and can be regarded as concepts of interest to the patients in relevant clinical trials [17]. For the cognitive debriefing, participants were asked to complete the Skindex-10 and were then asked about the instrument’s content and wording, response options, and recall period. A high-level qualitative analysis of the first five interviews determined that no modifications to the Skindex-10 were required. Content validity interviews were not conducted for the 5-D Itch scale because patients with kidney disease were included in the original development and validation of this instrument [14].

### Patient population and data collection

Psychometric properties of the Skindex-10 and 5-D Itch scale were assessed using data collected from a phase 2 randomized placebo-controlled multicenter study in the US investigating the safety and efficacy of intravenous difelikefalin in patients with moderate-to-severe pruritus undergoing hemodialysis [11]. Eligibility criteria for patients in the phase 2 (*N* = 174) trial are given elsewhere [11]. Briefly, patients had to be aged ≥ 18 years, on hemodialysis three times per week for ≥ 3 months before screening, self-reporting pruritus ≤ 1 month before screening, and have moderate-to-severe pruritus at screening (i.e., severity of >4 on the WI-NRS, calculated as the average of the daily WI-NRS scores collected over a 7-day run-in period).

Skindex-10 and 5-D Itch measures were scheduled to be completed by patients during the trial at baseline (Day 1, before first dose), Weeks 2, 4, 6, and the end of treatment (Week 8 or at study discontinuation). Other measures used in the phase 2 study (Self-Assessed Disease Severity [SADS] [3], MOS sleep scale [18], Patient Global Impression of Worst Itch Severity [PGI-S] [19], and Patient Global Impression of Change [PGI-C] [19]) were to be completed according to the schedule described in Table 1. All assessments were collected at the end of each respective treatment week, i.e., before dialysis and before administration of the next week’s dose.

**Table 1:** Schedule of study assessment activities

Study Procedures	Screen	Treatment period (Week)								End of treatment/ W8
	Day −14 to Day −1	Baseline^a^	W1	W2	W3	W4	W5	W6	W7	
SADS	X									
WI-NRS (daily)	X	X	X	X	X	X	X	X	X	X
Skindex-10		X		X		X		X		X
5-D Itch		X		X		X		X		X
MOS Sleep		X			X				X	X
PGI-S		X	X		X				X	X
PGI-C										X

Assessments were completed at the end of each treatment cycle, before dialysis (i.e., Week 1 = Day 8, Week 2 = Day 15, Week 3 = Day 22, Week 4 = Day 29, Week 5 = Day 36, Week 6 = Day 43, Week 7 = Day 50, End of Treatment/Week 8 = Day 57). Abbreviations: MOS, Medical Outcomes Study; PGI-C, Patient Global Impression of Change; PGI-S, Patient Global Impression of Worst Itch Severity; SADS, Self-Assessed Disease Severity; WI-NRS, Worst Itching Intensity Numerical Rating Scale

^a^ Baseline defined as Study Day 1 before first dose of study treatment

### Psychometric analyses

Psychometric assessments were analyzed in accordance with classical psychometric theory [20] and the US Food and Drug Administration guidance on PROs [7]. Statistical analyses were conducted in SAS version 9.4 and used a 2-sided significance level of *P* < 0.05. An overview of the anchor measures used for evaluating the measurement properties of the Skindex-10 and 5-D Itch scales is provided in Table 2.

**Table 2:** Anchor measures

Measure	Response scale	Recall period	References
**WI-NRS** (Worst Itching Intensity Numerical Rating Scale)	Patients rate their worst itch in the past 24 h on an 11-point scale: 0–10. “0” labelled with the anchor phrase “No itching” and “10” labelled “Worst itching imaginable”	24 h	[8, 9]
**SADS** (Self-Assessed Disease Severity)	Patients self-assess their pruritus severity by selecting one of three profiles based on occurrence of scratch marks on skin, problems sleeping due to itching, and feelings of agitation or sadness: Patient A (mild signs and symptoms), Patient B (moderate signs and symptoms), or Patient C (severe signs and symptoms)	Current	[3]
**PGI-C** (Patient Global Impression of Change)	Asks patients to indicate the change of their condition (improvement or worsening) from an earlier time point (e.g., vs. the start of a clinical trial). Single-item measure with categories ranging from “Very much improved” to “Very much worse”	Current vs. earlier time point	[19]
**PGI-S** (Patient Global Impression of Worst Itch Severity)	Asks patients to describe their worst itching in the past 24 h. Single-item scale with five possible values ranging from “None” to “Very severe”	24 h	[19]
**MOS Sleep Scale**	Measures various aspects of sleep, including sleep disturbance and adequacy. Most questions use a 6-point scale where “1” is labeled “All of the time” and “6” is labeled “None of the time”, indicating the frequency of various aspects of sleep disruption. Instructions are also provided to estimate the average hours of sleep during the past week and the length of time taken to fall asleep. Subscales that can be calculated include Sleep Problem Index II (9 items: 1, 3, 4, 5, 6, 7, 8, 9, 12), Sleep Problem Index I (6 items: 4, 5, 7, 8, 9, 12), and Sleep Disturbance (4 items: 1, 3, 7, 8). Higher scores reflect better sleep-related HRQoL.	Past week	[18]

Missing data were handled according to the instructions provided by the instrument authors. Abbreviations: HRQoL, health-related quality of life; MOS, Medical Outcomes Study

### Reliability

Internal-consistency reliability of Skindex-10 and 5-D Itch scores were estimated using Cronbach’s alpha coefficient, which is based on the number of items in a scale and the average inter-item correlation. Reliability coefficients of 0.90 or greater have been suggested for individual-level analyses [21] and an internal consistency of 0.80 is considered to be sufficient for group-level analysis [22].

Test-retest reliability for the Skindex-10 and 5-D Itch was assessed in subjects who were stable (i.e., had the same response) on the PGI-S between baseline and Week 1, or between Weeks 1 and 3, given the efficacy of difelikefalin establishes after these time points [10, 11]. Because the PGI-S was administered at a different schedule to the Skindex-10 and 5-D Itch in the clinical trial (see Table 1), test-retest reliability for the Skindex-10 and 5-D Itch was assessed at the closest time points available – between baseline and Week 2, and between Weeks 2 and 4. Intraclass correlation coefficients (ICCs) were based on the ICC(2,1) method [23]. ICCs were interpreted based on the recommendations of Koo and Li [24]: values less than 0.5 are indicative of poor reliability, values between 0.5 and 0.75 indicate moderate reliability, values between 0.75 and 0.9 indicate good reliability, and values greater than 0.9 indicate excellent reliability.

### Construct validity

Convergent validity is an evaluation of the relationship (correlation) between measures that assess roughly similar constructs. To evaluate convergent validity, the total and domain scores of the Skindex-10 and 5-D Itch were correlated with the WI-NRS, the PGI-S, and with one another at baseline and at end of treatment (Week 8). Moderate (*r* ≥ 0.3 to < 0.5) or large (*r* ≥ 0.5) convergent correlations by Cohen’s standards [25] were hypothesized between the Skindex-10, 5-D Itch, WI-NRS, and PGI-S measures. The MOS sleep measures were used for tests of divergent validity (i.e., to assess the extent to which sleep and itch, which are less related concepts, exhibit low correlations [*r* < 0.3] with one another). Pearson correlations were used and justified based on the results of the Kolmogorov-Smirnov test, which supported the assumption of distributional normality.

Known-groups validity is an additional method to provide evidence of construct validity. The known groups validity of the 5-D Itch scale and Skindex-10 questionnaire was assessed by forming known groups from the other PRO data collected in the original study (PGI-S, SADS, WI-NRS). The mean baseline and end-of-treatment 5-D Itch total scores and Skindex-10 total scores were then computed for each known group. WI-NRS known groups were based on established severity bands [26], derived from the weekly mean WI-NRS scores. As the data were normally distributed (by Kolmogorov-Smirnov test), two-sample t-tests were used to compare differences in 5-D Itch and Skindex-10 for known groups with two categories, and linear model ANOVA were used to compare differences for known groups with more than two categories (mean 5-D Itch or Skindex-10 score as the dependent variable and the categorical known group as the independent variable; separate models for each individual known group).

### MWPC threshold study & analysis

Anchor-based methods [8] were used to determine MWPC thresholds in Skindex-10 and 5-D Itch scores at the end of treatment. Briefly, these methods involved selecting and categorizing appropriate anchors, linking changes in the PRO measures to these anchors, and determining MWPC thresholds based on these relationships. Mean change scores, mean percentage change from baseline, and effect sizes were calculated to quantify the relationship between changes in the PRO measures and the anchor categories.

The PGI-C was selected as an anchor variable because it is commonly used to evaluate meaningful change, including within pruritus populations [8, 9, 27], and is recommended by the US Food and Drug Administration as a suitable anchor [28]. The PGI-C asks patients to indicate the change in itch (i.e., since the start of the study) taking into consideration treatment effect and patient expectation. To mitigate potential recall bias with the PGI-C, we also incorporated the PGI-S (also suggested by the US Food and Drug Administration [28]) as an alternative anchor to define clinically meaningful change. The PGI-S asks patients to choose the option that best describes their worst itching in the past 24 h on a scale from 0 (None) to 4 (Very Severe). The “minimally improved” and combined “minimally or much improved” PGI-C categories, and a one-point categorical severity change on the PGI-S, were the primary anchors used to define meaningful improvements on the Skindex-10 and 5-D Itch. A one-point change on the PGI-S (e.g., from “moderate” to “mild”) was considered clinically meaningful. This determination is supported by epidemiological data showing subjects with severe itch (≥ 7 on a 0 to 10-point scale) have a higher mortality rate than patients with moderate (4.0 to 6.9) or mild (< 4.0) itch [1]. As a sensitivity analysis, the “much improved” PGI-C category and a ≥ 1-point categorical severity change on the PGI-S were used as anchors corresponding with larger improvements in patients’ conditions.

To verify the MWPC thresholds, the same method using the PGI-C anchor was applied to two similar, larger phase 3 study populations of patients with moderate-to-severe CKD-aP undergoing hemodialysis (KALM-1, *N* = 378 and KALM-2, *N* = 471). The trial designs, eligibility criteria, demographics, and baseline clinical characteristics were very similar between the two phase 3 cohorts, and also very similar to those of the phase 2 cohort, with the exception that KALM-2 was a global trial (rather than being purely US-based) and included a lower proportion of patients who were Black or African American [10, 13, 29].

## Results

### Skindex-10 content validity

The content validity cohort (*N* = 23) had a median age of 61.0 years (range, 25.0–82.0), most were male (60.9%), 43.5% were white, and 26.1% were Black or African American. The cohort had been on hemodialysis for a mean of 5.4 years (standard deviation [SD], 5.1; further demographic and clinical characteristics of the cohort are published elsewhere [9]).

Full details of the concept elicitation portion of the 23 interviews have been previously reported [17]. In summary, the qualitative interviews showed that most patients with CKD-aP experienced daily itching (*n* = 12, 52.2%), which was often more severe at night and during or after dialysis, significantly affecting their quality of life and daily activities. Participants described various itching symptoms, with common areas affected including the back, arms, head, and legs. All 23 participants (100%) discussed feeling bothered or impacted by their itching; the most reported HRQoL impacts included difficulties carrying out daily activities (e.g., selecting clothes to wear, bathing, running errands) (*n* = 20, 87.0%), impact on sleep (*n* = 19, 82.6%), and emotional/psychological impacts (*n* = 9, 39.1%).

Overall, participants provided positive feedback on the Skindex-10, and reported that the instrument was straightforward, comprehensive, and relevant to their experience with CKD-aP. In addition, most participants indicated the instructions, item wording, and response options were easy to understand; participants were able to easily select a response option and describe how they arrived at their answers. Based on a detailed review of the data, no changes to the Skindex-10 content were recommended. However, it was determined necessary that study staff emphasize the recall period for future patients, because some participants did not independently think about the stated recall period when choosing an answer (although they understood the recall period when asked).

### Psychometric validation

Demographics of the phase 2 cohort (*N* = 174) are published elsewhere [9]. Briefly, this US cohort had a median age of 59.0 years (range, 26.0–84.0), most were male (60.3%), 58.6% were Black or African American, and 20.7% were Hispanic or Latino. The cohort had been on hemodialysis for a mean of 5.8 years (SD, 4.7).

### Internal consistency reliability

Internal consistency reliability was strong for the Skindex-10 total score (Cronbach’s α ≥ 0.9) and moderate-to-strong for the Skindex-10 domains at both time points (Cronbach’s α ≥ 0.76; Table 3). Internal consistency reliability was moderate for the 5-D Itch total score (Cronbach’s α, 0.74 at both time points).

**Table 3:** Internal consistency reliability

Measure	Time point	*N*	Cronbach’s α
**Skindex-10**			
Total score	Baseline	170	0.90
	Week 8	146	0.95
Disease domain	Baseline	172	0.76
	Week 8	149	0.86
Mood/emotional distress	Baseline	173	0.80
	Week 8	147	0.92
Social functioning	Baseline	172	0.90
	Week 8	148	0.95
**5-D Itch**			
Total score	Baseline	174	0.74
	Week 8	145	0.74

5-D Itch total score is the sum of duration, degree, direction, disability, and distribution. Skindex-10 total score is the sum of the 10 items

### Test-retest reliability

Patients that were stable on the PGI-S had moderate reproducibility of Skindex-10 total scores between baseline and Week 2, and between Week 2 and Week 4 (ICC values >0.7; Table 4), according to the interpretation recommendations by Koo and Li [24]. Patients stable on the PGI-S had moderate reproducibility of 5-D Itch total scores between Week 2 and Week 4 (ICC = 0.67), and reproducibility just below the threshold for moderate between baseline and Week 2 (ICC = 0.48).

**Table 4:** Test-retest reliability

Test-retest time points	Mean (SD)	Difference in means (SD)	T-value	*P*-value	ICC (95% CI)
**Skindex-10 (total score)**					
Baseline–Week 2 (*N* = 62)^a^					
Baseline	32.6 (12.6)	–	–	–	–
Week 2	28.9 (14.3)	−3.7 (9.68)	−3.04	0.0034	0.717 (0.573, 0.818)
Week 2–Week 4 (*N* = 64)^b^					
Week 2	27.8 (14.8)	–	–	–	–
Week 4	23.2 (13.9)	−4.6 (10.5)	−3.50	0.0009	0.701 (0.554, 0.806)
**5-D Itch (total score)**					
Baseline–Week 2 (*N* = 64)^a^					
Baseline	16.1 (3.17)	–	–	–	–
Week 2	14.7 (3.55)	−1.5 (3.28)	−3.54	0.0007	0.484 (0.275, 0.649)
Week 2–Week 4 (*N* = 67)^b^					
Week 2	13.8 (3.60)	–	–	–	–
Week 4	12.8 (3.79)	−1.0 (2.93)	−2.71	0.0086	0.666 (0.510, 0.780)

Test-retest reliability for subgroups of the phase 2 cohort (*N* = 174) with stable PGI-S scores. Skindex-10 total score is the sum of the 10 items. 5-D Itch total score is the sum of duration, degree, direction, disability, and distribution. Abbreviations: ICC, intraclass correlation coefficient; PGI-S, Patient Global Impression of Worst Itch Severity; SD, standard deviation

^a^ For patients with stable PGI-S between baseline and Week 1

^b^ For patients with stable PGI-S between Week 1 and Week 3

### Convergent and divergent validity

Skindex-10 total scores showed moderate-to-large convergent correlations with the WI-NRS, 5-D Itch total scores, and PGI-S at baseline (*r* = 0.32–0.65, *P*-values < 0.0001) and large correlations with these measures at end of treatment (*r* = 0.58–0.74, *P*-values < 0.0001; Table 5). Skindex-10 total scores and the “disease” and “mood/emotional distress” domains correlated especially with WI-NRS scores and the conceptually related 5-D Itch “degree” and “disability” domains at the end of treatment (*r* = 0.59–0.70). 5-D Itch total score and the “degree” and “disability” domains showed moderate-to-large convergent correlations with the WI-NRS (*r* = 0.30–0.71; *P* -values < 0.0001) and large convergent correlations with the PGI-S at both time points (*r* = 0.51–0.74; *P* -values < 0.0001; Table 6). For both the Skindex-10 and 5-D Itch, correlations were generally stronger with conceptually related measures at the end of treatment than at baseline, likely due to higher score variance at end of treatment (to be randomized, subjects had to report WI-NRS > 4 at screening).

As hypothesized, correlations with the conceptually unrelated domains of the MOS Sleep measure (“Sleep Problem Index I and II”, and “Sleep Disturbance”) were small to moderate (*r* = 0.12–0.48) by Cohen’s standards [25], at both time points (Tables 5 and 6).

**Table 5:** Skindex-10: convergent and divergent validity

Timepoint	Comparator measure	Pearson correlations between Skindex-10 and related/unrelated measures (r-value)			
		Skindex-10 Total score	Skindex-10 Disease domain	Skindex-10 Mood/ emotional distress	Skindex-10 Social functioning
**Baseline**	WI-NRS	0.32^****^	0.34^****^	0.35^****^	0.21^**^
	5-D Itch				
	Total score	0.65^****^	0.57^****^	0.62^****^	0.51^****^
	Degree	0.51^****^	0.47^****^	0.51^****^	0.39^****^
	Duration	0.51^****^	0.47^****^	0.49^****^	0.39^****^
	Direction	0.39^****^	0.45^****^	0.40^****^	0.26^***^
	Disability	0.55^****^	0.41^****^	0.51^****^	0.47^****^
	Distribution	0.34^****^	0.27^***^	0.30^****^	0.31^****^
	PGI-S	0.44^****^	0.44^****^	0.43^****^	0.32^****^
	MOS Sleep				
	Sleep Problem Index I	0.32^****^	0.15	0.29^***^	0.32^****^
	Sleep Problem Index II	0.33^****^	0.17^*^	0.32^****^	0.32^****^
	Sleep Disturbance	0.26^***^	0.13	0.30^****^	0.23^**^
**End of treatment (Week 8)**	WI-NRS	0.67^****^	0.80^****^	0.61^****^	0.48^****^
	5-D Itch				
	Total score	0.74^****^	0.74^****^	0.69^****^	0.60^****^
	Degree	0.60^****^	0.64^****^	0.59^****^	0.43^****^
	Duration	0.55^****^	0.44^****^	0.49^****^	0.53^****^
	Direction	0.39^****^	0.53^****^	0.36^****^	0.23^**^
	Disability	0.69^****^	0.61^****^	0.70^****^	0.58^****^
	Distribution	0.44^****^	0.41^****^	0.36^****^	0.40^****^
	PGI-S	0.58^****^	0.62^****^	0.57^****^	0.41^****^
	MOS Sleep				
	Sleep Problem Index I	0.24^**^	0.19^*^	0.26^**^	0.23^**^
	Sleep Problem Index II	0.29^***^	0.26^**^	0.29^***^	0.25^**^
	Sleep Disturbance	0.26^**^	0.27^***^	0.25^**^	0.22^**^

Pearson correlations: * *P* < 0.05, ** *P* < 0.01, *** *P* < 0.001, **** *P* < 0.0001. Abbreviations: MOS, Medical Outcomes Study; PGI-S, Patient Global Impression of Worst Itch Severity; WI-NRS, Worst Itching Intensity Numerical Rating Scale

**Table 6:** 5-D Itch: convergent and divergent validity

Timepoint	Comparator measure	Pearson correlations between 5-D Itch and related/unrelated measures (r-value)					
		5-D Itch Total score	Degree domain	Duration domain	Direction domain	Disability domain	Distribution domain
**Baseline**	WI-NRS	0.31^****^	0.30^****^	0.22^**^	0.12	0.33^****^	0.14
	PGI-S	0.62^****^	0.70^****^	0.43^****^	0.39^****^	0.55^****^	0.28^***^
	MOS Sleep						
	Sleep Problem Index I	0.40^****^	0.33^****^	0.24^**^	0.23^**^	0.48^****^	0.16^*^
	Sleep Problem Index II	0.44^****^	0.37^****^	0.26^***^	0.31^****^	0.48^****^	0.21^**^
	Sleep Disturbance	0.36^****^	0.33^****^	0.22^**^	0.26^***^	0.42^****^	0.12
**End of treatment (Week 8)**	WI-NRS	0.71^****^	0.67^****^	0.46^****^	0.51^****^	0.56^****^	0.37^****^
	PGI-S	0.70^****^	0.74^****^	0.43^****^	0.55^****^	0.51^****^	0.29^***^
	MOS Sleep						
	Sleep Problem Index I	0.33^****^	0.21^*^	0.27^***^	0.15	0.36^****^	0.19^*^
	Sleep Problem Index II	0.39^****^	0.23^**^	0.30^***^	0.21^*^	0.39^****^	0.22^**^
	Sleep Disturbance	0.34^****^	0.17^*^	0.25^**^	0.23^**^	0.37^****^	0.17^*^

Pearson correlations: *P < 0.05, ** *P* < 0.01, *** *P* < 0.001, **** *P* < 0.0001. Abbreviations: MOS, Medical Outcomes Study; PGI-S, Patient Global Impression of Worst Itch Severity; WI-NRS, Worst Itching Intensity Numerical Rating Scale

### Known-groups validity

Skindex-10 total scores at baseline and end of treatment (Week 8) were significantly different (*P* ≤ 0.0015) and in the anticipated direction between known groups of the conceptually related PGI-S and SADS (‘Profile B’ versus ‘Profile C’) measures, and at end of treatment when grouped by WI-NRS severity bands (Table 7). The same was true for 5-D Itch total scores between the known groups of these measures (*P* ≤ 0.0017; Table 8). Overall, higher (worse) mean Skindex-10 and 5-D Itch total scores were observed for groups with worse categories defined by these independent measures.

**Table 7:** Skindex-10: known-groups validity at baseline and end of treatment^a^

Comparator measure	*N*	Mean Skindex-10 total score (SD)	T-value	F-value	*P*-value
**SADS**					
Baseline	170		−3.23	–	0.0015
Profile B (moderate)	120	32.1 (11.4)			
Profile C (severe)	50	38.7 (13.8)			
**PGI-S**					
Baseline	170		–	13.60	< 0.0001
None	0	– (–)			
Mild	7	23.4 (12.2)			
Moderate	76	29.3 (11.3)			
Severe	73	38.0 (10.7)			
Very severe	14	44.6 (13.7)			
End of treatment	146		–	21.97	< 0.0001
None	8	1.1 (1.36)			
Mild	62	12.9 (8.49)			
Moderate	54	26.8 (13.9)			
Severe	21	29.1 (14.6)			
Very severe	1	60.0 (–)			
**WI-NRS**					
Baseline	167		–	6.15	0.0027
None (0)	0	– (–)			
Mild (> 0 to < 4)	25	30.2 (7.86)			
Moderate (≥ 4 to < 7)	91	32.5 (12.6)			
Severe (≥ 7 to 10)	51	39.0 (13.0)			
End of treatment	141		–	24.60	< 0.0001
None (0)	4	2.3 (3.30)			
Mild (> 0 to < 4)	67	13.3 (10.1)			
Moderate (≥ 4 to < 7)	49	23.0 (12.3)			
Severe (≥ 7 to 10)	21	36.4 (15.9)			

Differences in Skindex-10 total scores by known groups were evaluated by linear model ANOVA or t-test. ANOVA *P*-values are for overall heterogeneity. Abbreviations: PGI-S, Patient Global Impression of Worst Itch Severity; SADS, Self-Assessed Disease Severity; SD, standard deviation; WI-NRS, Worst Itching Intensity Numerical Rating Scale

^a^ End of treatment = Week 8

**Table 8:** 5-D Itch: known-groups validity at baseline and end of treatment^a^

Comparator measure	*N*	Mean 5-D Itch total score (SD)	T-value	F-value	*P*-value
**SADS**					
Baseline	174		−3.19	–	0.0017
Profile B (moderate)	123	16.3 (3.24)			
Profile C (severe)	51	18.2 (3.76)			
**PGI-S**					
Baseline	174		–	27.84	< 0.0001
None	1	6.00 (–)			
Mild	8	12.8 (3.65)			
Moderate	77	15.3 (2.74)			
Severe	74	18.4 (2.62)			
Very severe	14	20.6 (2.84)			
End of treatment	149		–	38.03	< 0.0001
None	8	6.1 (2.17)			
Mild	63	10.7 (2.24)			
Moderate	56	13.7 (3.01)			
Severe	21	16.2 (2.86)			
Very severe	1	25.0 (–)			
**WI-NRS**					
Baseline	171		–	3.73	0.0261
None (0)	0	– (–)			
Mild (> 0 to < 4)	25	16.4 (3.49)			
Moderate (≥ 4 to < 7)	94	16.4 (3.43)			
Severe (≥ 7 to 10)	52	18.0 (3.46)			
End of treatment	143		–	32.92	< 0.0001
None (0)	4	6.0 (2.45)			
Mild (> 0 to < 4)	67	10.5 (2.55)			
Moderate (≥ 4 to < 7)	51	13.7 (3.04)			
Severe (≥ 7 to 10)	21	16.2 (3.31)			

Differences in 5-D Itch total scores by known groups were evaluated by linear model ANOVA or t-test. ANOVA *P*-values are for overall heterogeneity. Abbreviations: PGI-S, Patient Global Impression of Worst Itch Severity; SADS, Self-Assessed Disease Severity; SD, standard deviation; WI-NRS, Worst Itching Intensity Numerical Rating Scale

^a^ End of treatment = Week 8

### MWPC thresholds

The mean change in Skindex-10 total score associated with a change from baseline to “minimally improved” on the PGI-C was −18.5 points for the Phase 2 cohort (47.4% change; Table 9). However, in the two larger Phase 3 cohorts, the change was smaller in magnitude (−12.4 and −10.6 points; 14.4%–27.4% change). The mean change associated with a change to “minimally or much improved” on the PGI-C was consistent between the three cohorts, ranging between −13.8 and −15.8 points (27.0%–41.0% change). The mean change in Skindex-10 total score associated with a one-point improvement on the PGI-S was −13.3 points (38.6% change).

**Table 9:** MWPC thresholds for Skindex-10 by PGI-C category and PGI-S score change

	*N*	Skindex-10 total score change^a^		Mean % change from baseline	Effect Size (Cohen’s d)
		Mean (SD)	Median		
**PGI-C**					
Minimally improved					
* Phase 2 cohort*	23	−18.5 (12.4)	−16.0	−47.4	−1.49
* Phase 3 cohort 1*	91	−12.4 (13.8)	−11.0	−27.4	−0.82
* Phase 3 cohort 2*	115	−10.6 (14.2)	−10.0	−14.4	−0.73
Much improved					
* Phase 2 cohort*	51	−14.2 (13.0)	−13.0	−37.5	−1.10
* Phase 3 cohort 1*	98	−19.0 (13.7)	−17.0	−53.5	−1.39
* Phase 3 cohort 2*	108	−17.2 (14.8)	−18.0	−40.3	−1.24
Minimally or much improved					
* Phase 2 cohort*	74	−15.6 (12.9)	−14.0	−40.5	−1.21
* Phase 3 cohort 1*	189	−15.8 (14.1)	−14.0	−41.0	−1.06
* Phase 3 cohort 2*	223	−13.8 (14.9)	−14.0	−27.0	−0.94
No change					
* Phase 2 cohort*	25	−2.5 (9.9)	−3.0	−7.6	−0.25
* Phase 3 cohort 1*	46	−2.5 (13.6)	−5.0	−2.9	−0.15
* Phase 3 cohort 2*	84	−5.6 (13.2)	−4.0	−13.4	−0.37
**PGI-S (Phase 2 cohort)**					
Improved one point	56	−13.3 (13.0)	−14.0	−38.6	−1.03
Improved at least one point	97	−18.6 (14.1)	−18.0	−51.6	−1.31
No change	36	−7.7 (11.2)	−6.0	−20.9	−0.69

Abbreviations: MWPC, meaningful within-patient change; PGI-C, Patient Global Impression of Change; PGI-S, Patient Global Impression of Worst Itch Severity; SD, standard deviation

^a^ Change from end of treatment (Week 8) to baseline

The mean change in 5-D Itch total score associated with a change from baseline to “minimally improved” on the PGI-C was −4.4 points (24.6% change) for the Phase 2 cohort and −3.5 points (18.7%–19.1% change) for the two Phase 3 validation cohorts (Table 10). The mean total score change associated with a change to “minimally or much improved” on the PGI-C ranged between −4.2 and −4.7 points (23.8%–26.0% change) for the three cohorts. The mean change in 5-D Itch total score associated with a one-point improvement on the PGI-S for the Phase 2 cohort was −4.1 points (23.4% change).

**Table 10:** MWPC thresholds for 5-D Itch by PGI-C category and PGI-S score change

	*N*	5-D Itch total score change^a^		Mean % change from baseline	Effect Size (Cohen’s d)
		Mean (SD)	Median		
**PGI-C**					
Minimally improved					
* Phase 2 cohort*	25	−4.4 (3.0)	−4.0	−24.6	−1.45
* Phase 3 cohort 1*	96	−3.5 (3.3)	−3.0	−18.7	−1.04
* Phase 3 cohort 2*	116	−3.5 (3.7)	−3.0	−19.1	−1.08
Much improved					
* Phase 2 cohort*	51	−4.7 (3.3)	−4.0	−25.4	−1.42
* Phase 3 cohort 1*	102	−5.8 (3.5)	−5.0	−32.8	−1.84
* Phase 3 cohort 2*	111	−4.8 (3.3)	−5.0	−28.7	−1.52
Minimally or much improved					
* Phase 2 cohort*	76	−4.6 (3.2)	−4.0	−25.1	−1.43
* Phase 3 cohort 1*	198	−4.7 (3.6)	−4.0	−26.0	−1.38
* Phase 3 cohort 2*	227	−4.2 (3.6)	−4.0	−23.8	−1.24
No change					
* Phase 2 cohort*	25	−0.3 (2.0)	−1.0	−1.4	−0.16
* Phase 3 cohort 1*	47	−0.8 (3.1)	−1.0	−2.7	−0.21
* Phase 3 cohort 2*	90	−1.14 (3.3)	−1.0	−5.1	−0.32
**PGI-S (Phase 2 cohort)**					
Improved one point	57	−4.1 (3.2)	−4.0	−23.4	−1.29
Improved at least one point	100	−6.0 (4.1)	−6.0	−33.0	−1.49
No change	37	−2.6 (2.9)	−3.0	−14.7	−0.89

Abbreviations: MWPC, meaningful within-patient change; PGI-C, Patient Global Impression of Change; PGI-S, Patient Global Impression of Worst Itch Severity; SD, standard deviation

^a^ Change from end of treatment (Week 8) to baseline

## Discussion

Although several PRO measures are available to measure itch and its impact on patients’ quality of life, few have undergone full psychometric validation in patients with CKD-aP [9]. The Skindex-10 questionnaire was specifically developed for CKD-aP in patients undergoing hemodialysis, but underwent only a preliminary psychometric validation in an observational study cohort (*N* = 103), in which it showed high correlation with other PROs of itch intensity and HRQoL [3]. The 5-D Itch was originally validated in five groups of patients, each with a different form of chronic pruritus. Convergent validity was measured against itching severity recorded on a visual analogue scale and the PBC-40 quality of life assessment of pruritus associated with primary biliary cirrhosis [14]. Here, building on these initial studies, we confirmed that the Skindex-10 questionnaire and the 5-D Itch scale have desirable psychometric properties as PRO measures for patients with moderate-to-severe CKD-aP and provide MWPC thresholds (or ‘responder definitions’) for this population.

The patient interviews provided qualitative support for the content validity of the Skindex-10. Patients found that the instrument’s content was important, comprehensive, and relevant to their experience with CKD-aP, and most confirmed that its instructions, item wording, and response options were easy to understand. Internal consistency reliability was strong for the Skindex-10 and moderate for the 5-D Itch. Both instruments showed moderate test-retest reliability based on patients with stable PGI-S values, and both instruments – especially their conceptually related domains – showed moderate-to-strong correlations with WI-NRS and PGI-S scores at the end of treatment. Similarly, the Skindex-10 and 5-D Itch total scores correlated to known groups of conceptually related measures.

For the Skindex-10, we suggest a ≥ 15-point change in Skindex-10 total score to be a suitable responder definition indicative of MWPC for populations with moderate-to-severe CKD-aP. This threshold is close to the mean score change of 15.8 for the three primary anchor-based estimates for the Phase 2 cohort (PGI-C “minimally improved”, PGI-C “minimally or much improved”, and PGI-S “improved one point”). We choose to round down to 15 because the score-change estimates for PGI-C “minimally improved” in the two larger Phase 3 cohorts were considerably lower (−12.4 and −10.6 points; 14.4%–27.4% change) than that for the Phase 2 cohort (−18.5; 47.4% change). Additionally, the score-change estimates for the combined PGI-C “minimally or much improved” categories were more consistent across the three cohorts, with a mean score change of −15.1.

For the 5-D Itch total score, we suggest a ≥ 5-point change be used as a suitable responder definition indicative of a MWPC for populations with moderate-to-severe CKD-aP. This threshold aligns with the mean for the three primary anchor-based estimates of 4.4 and is supported by the anchor-based analysis of the two larger phase 3 cohorts.

This study has limitations. The results may not fully represent the real-world population as the patients included had to meet the eligibility criteria of the original clinical trials – one of those criteria being the participants having moderate-to-severe CKD-aP. Many patients with CKD-aP experience milder itching [2–4], therefore the MWPC thresholds determined in this study may not be appropriate for all patients. In addition, due to the design of the trials, the time points for data collection on the different measures did not always coincide. This discrepancy potentially added variability and may have resulted in an underestimation of the ICCs in the test-retest reliability analyses. Finally, a further limitation is that the demographics of the cohorts used were slightly skewed towards males.

The Skindex-10 questionnaire and 5-D Itch scale have been recently used to assess endpoints in clinical trials evaluating medications for CKD-aP and other forms of pruritus [10, 30, 31]. While these comprehensive PRO measures are valuable in clinical research, they may not be as practical or effective in everyday clinical practice. In this setting, simpler methods, such as a single itch-related question [32], have been explored to facilitate routine assessments of patients receiving dialysis and to reduce the underdiagnosis of CKD-aP.

## Conclusions

The results from this study suggest that the Skindex-10 questionnaire and 5-D Itch scale have good psychometric properties and are effective in measuring itch intensity and HRQoL in patients with moderate-to-severe CKD-aP receiving dialysis. The proposed MWPC thresholds of a ≥ 15-point reduction on the Skindex-10 questionnaire and a ≥ 5-point reduction on the 5-D Itch scale can be used to provide an a priori responder definition to assess treatment benefit in clinical trials involving this patient population.

## Supplementary Information

Below is the link to the electronic supplementary material.

Supplementary Material 1

## Data Availability

The datasets generated and analysed during the current study are not publicly available due to privacy or ethical restrictions but are available from the corresponding author on reasonable request.

## Abbreviations

CKD: Chronic kidney disease
CKD-aP: Chronic kidney disease-associated pruritus
HRQoL: Health-related quality of life
ICC: Intraclass correlation coefficient
MOS: Medical Outcomes Study
NS: Not significant
NRS: Numerical rating scale
PGI: Patient Global Impression
PGI-C: Patient Global Impression of Change
PGI-S: Patient Global Impression of Worst Itch Severity
PRO: Patient-reported outcome
SADS: Self-Assessed Disease Severity
SD: Standard deviation
WI-NRS: Worst Itching Intensity numeric rating scale
